# Predicting difficult laryngoscopy via three-dimensional graphical modeling and hybrid artificial intelligence using standard facial photographs in elective surgery patients

**DOI:** 10.1186/s12871-026-04160-9

**Published:** 2026-08-15

**Authors:** Bahar Uslu Bayhan, Ilkay Sibel Kervancı, Tuğçe Gazioğlu Kişi, Burak Cemil Balık, Medine Atmaca, Kazim Ersin Altinsoy

**Affiliations:** 1 Department of Anesthesiology and Reanimation, Gaziantep City Hospital, Gaziantep, Türkiye; 2 Department of Computer Engineering, Faculty of Engineering, Gaziantep University, Gaziantep, Türkiye; 3 Department of Mathematics, Graduate School of Natural and Applied Sciences, Gaziantep University, Gaziantep, Türkiye; 4 Department of Emergency Medicine, Faculty of Medicine, Gaziantep Islam Science and Technology University, Gaziantep, Türkiye

**Keywords:** Preoperative Airway Management, difficult laryngoscopy, Anesthetic Patient Safety, Hybrid Ensemble Learning, Geometric Deep Learning, Explainable Artificial Intelligence

## Abstract

**Background:**

Unexpected difficult laryngoscopy remains one of the leading causes of life-threatening complications in anesthetic practice. Conventional airway assessment tests (e.g., Mallampati classification, thyromental distance) are inherently subjective and demonstrate limited diagnostic accuracy. We aimed to develop a hybrid artificial intelligence model that extracts three-dimensional geometric ratios from standardized facial photographs using deep learning and graph-based modeling to predict difficult laryngoscopy.

**Methods:**

In this prospective observational study, consecutive patients scheduled for elective surgery were enrolled. Preoperatively, standardized facial photographs were obtained from eight projection angles. Instead of raw pixel-based image analysis, the images were transformed into three-dimensional anatomical graph structures following local illumination normalization using Contrast Limited Adaptive Histogram Equalization (CLAHE), combined with MediaPipe Face Mesh (468 landmark points) and Pose Estimation (33 skeletal key points). Clinically relevant anatomical indices, including Retrognathia, Overjet, Neck Bulk, and Airway Visibility, were mathematically derived from these coordinates and combined with routinely collected clinical and anthropometric variables as structured model inputs. Cormack–Lehane (CL) grading was used as the reference standard (Grades I–II: non-difficult laryngoscopy; Grades III–IV: difficult laryngoscopy). The structured dataset was used to train XGBoost, Random Forest, and Tabular Transformer models incorporating multi-head attention. To prevent data leakage, Synthetic Minority Over-sampling Technique (SMOTE) augmentation and scaling were applied exclusively to the training set.

**Results:**

Preoperative images and anatomical ratios of 198 patients were analyzed. Among the derived indices, the Mouth Opening Ratio and Oropharyngeal Visibility Index showed the highest landmark acquisition success, with extraction in 193 of 198 patients (97.5%). The proposed hybrid artificial intelligence (AI) model achieved a test-set accuracy of 80.0%, sensitivity (recall) of 85.7%, and an area under the curve (AUC) of 0.808 for difficult laryngoscopy prediction.

**Conclusions:**

Unlike conventional convolutional neural networks (CNNs) relying on raw pixel processing, the proposed model combines standardized pixel-space measurements and normalized geometric ratios. This landmark-based representation may reduce sensitivity to some acquisition-related variability under standardized imaging conditions. These findings support the feasibility of a landmark-based, geometry-driven approach for objective preoperative airway risk assessment. However, external validation and direct comparison with standardized bedside airway assessment are required before routine clinical implementation.

**Supplementary Information:**

The online version contains supplementary material available at https://doi.org/10.1186/s12871-026-04160-9.

## Introduction

Tracheal intubation is a fundamental component of airway management during general anesthesia. Despite advances in anesthetic practice, unexpected difficult airway management remains an important cause of perioperative morbidity and mortality. Accurate preoperative identification of patients at risk of difficult intubation is therefore essential for appropriate preparation and airway management planning. The reported incidence of difficult intubation ranges from approximately 1% to 10% in operating room settings, with even higher rates in emergency and critical care environments [1, 2]. 

Several bedside airway assessment tests, including the Mallampati classification, thyromental distance, sternomental distance, upper lip bite test, and interincisor distance, are routinely used to estimate intubation difficulty. However, the predictive performance of these tests remains limited, with reported area under the receiver operating characteristic curve (AUC) values generally ranging between 0.60 and 0.70 when used individually. Their diagnostic accuracy is influenced by subjective assessment, patient cooperation, and the inability to comprehensively characterize individual anatomical variation, highlighting the need for more objective and reproducible approaches to airway assessment [1, 3–6]. 

Recent advances in artificial intelligence (AI), particularly deep learning and machine learning, have enabled automated analysis of medical images for diagnostic prediction. Several investigators have explored facial photograph–based prediction of difficult intubation using convolutional neural network (CNN) architectures. However, conventional CNN-based approaches rely on raw pixel information and are therefore susceptible to variations in lighting conditions, camera position, background, and image scale, potentially limiting their robustness in routine clinical practice [3, 7–10]. 

Recent narrative reviews further highlight the growing role of AI in airway management, including facial image analysis, multiparametric risk assessment, and real-time airway guidance, while emphasizing the importance of developing clinically reliable and interpretable prediction models [11]. 

The aim of the present study was to develop and internally validate a hybrid machine-learning model capable of predicting difficult laryngoscopy (Cormack–Lehane Grade III–IV) using routinely collected clinical and anthropometric variables together with anatomical landmark–derived geometric features extracted from standardized preoperative facial photographs. In the present study, the term hybrid refers to the weighted integration of complementary XGBoost and Tabular Transformer architectures and to the use of a unified structured input space comprising both clinical and photograph-derived variables. We hypothesized that this multimodal, geometry-informed hybrid approach would provide an objective method for predicting difficult laryngoscopy while reducing the limitations associated with conventional raw pixel-based image analysis.

## Materials and methods

The present study was designed and reported in accordance with the TRIPOD + AI statement for clinical prediction models that use machine learning methods, ensuring transparency and reproducibility of all modelling decisions [12].

This prospective, observational, single-center clinical study was conducted following approval from the Gaziantep City Hospital Non-Interventional Clinical Research Ethics Committee on April 15, 2026, under approval/protocol number 469/2026. Patients scheduled to undergo elective surgical procedures requiring general anesthesia at the Anesthesiology and Reanimation Clinic of Gaziantep City Hospital were assessed for eligibility. Written informed consent was obtained from all enrolled participants before study-related procedures.

### Inclusion criteria

- Aged 18 years and older,
- Scheduled for elective surgery requiring tracheal intubation under general anesthesia,
- Voluntary agreement to participate in the study.

### Exclusion criteria

- Patients with significant maxillofacial trauma, tumors, or severe anatomical deformities,
- Patients with cervical spine instability or clinically significant restriction of cervical movement that precluded safe completion of the standardized positioning maneuvers,
- Pregnant patients,
- Patients with facial hair or facial accessories interfering with airway assessment,
- Cases requiring emergency surgical intervention,
- Patients who declined participation or did not provide written informed consent.
- Intubations performed by physicians with less than 5 years of anesthesia experience.

During the routine preoperative anesthetic evaluation, conventional clinical airway assessment parameters—including Mallampati classification, thyromental distance, sternomental distance, and maximum interincisor opening—were recorded for all patients in accordance with established difficult airway management guidelines [13, 14]. Maximum interincisor opening was categorized as normal (≥ 4.0 cm), mildly to moderately restricted (3.0 to < 4.0 cm), or severely restricted (< 3.0 cm). For the binary variable INCIS_TEETH, values < 4.0 cm were classified as restricted. Neck movement was classified as restricted (NECK_MOVE) when either the patient was unable to touch the chin to the chest or cervical extension from the neutral position was < 30°. This functional classification was applied only to otherwise eligible patients who could safely complete the assessment maneuvers; the exclusion criterion referred to pre-existing or clinically significant cervical restriction that precluded safe standardized positioning. The upper lip bite test was also routinely assessed according to the institutional preoperative airway assessment protocol.

Laryngoscopy was performed using a standard Macintosh laryngoscope blade (No. 3 or 4) by an anesthesiologist with at least 5 years of clinical experience who was fully blinded to both the study protocol and the outputs of the artificial intelligence model. Airway visualization was graded according to the Cormack–Lehane (CL) classification system. Accordingly, CL Grades I–II were categorized as non-difficult laryngoscopy, whereas CL Grades III–IV were categorized as difficult laryngoscopy and served as the predefined binary reference outcome (ground truth) for model development. Although CL grade reflects laryngoscopic view rather than the overall clinical difficulty of intubation, it was selected because of its widespread use in airway prediction research and to facilitate comparison with previously published studies.

**Fig. 1:**
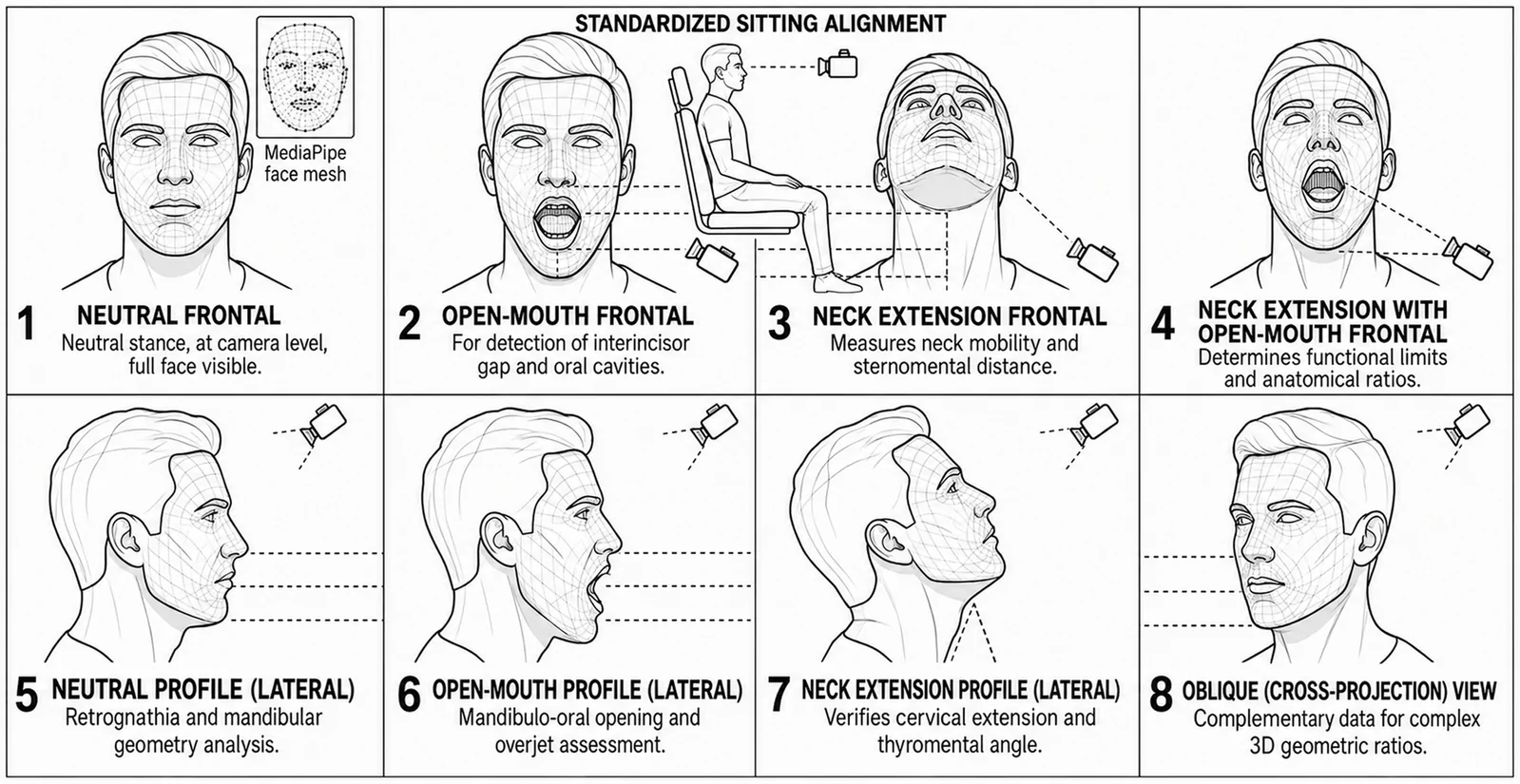
Standardized protocol for facial image acquisition. Eight predefined imaging positions were obtained under standardized lighting conditions and a fixed camera distance. A single high-resolution photograph was acquired for each position according to the predefined acquisition protocol

During the preoperative period, high-resolution facial photographs were acquired using the rear camera of an Apple iPhone 15 Pro Max smartphone (Apple Inc., Cupertino, CA, USA) under standardized lighting conditions and from a fixed distance, using eight predefined imaging positions (including neutral frontal view, frontal view with mouth opening, lateral profile view, and neck extension view). A single high-resolution photograph was obtained for each predefined imaging position according to the standardized acquisition protocol (Fig. 1). To ensure image standardization, all patients were instructed to remain seated in an upright position and look directly toward the camera during image acquisition.

**Fig. 2:**
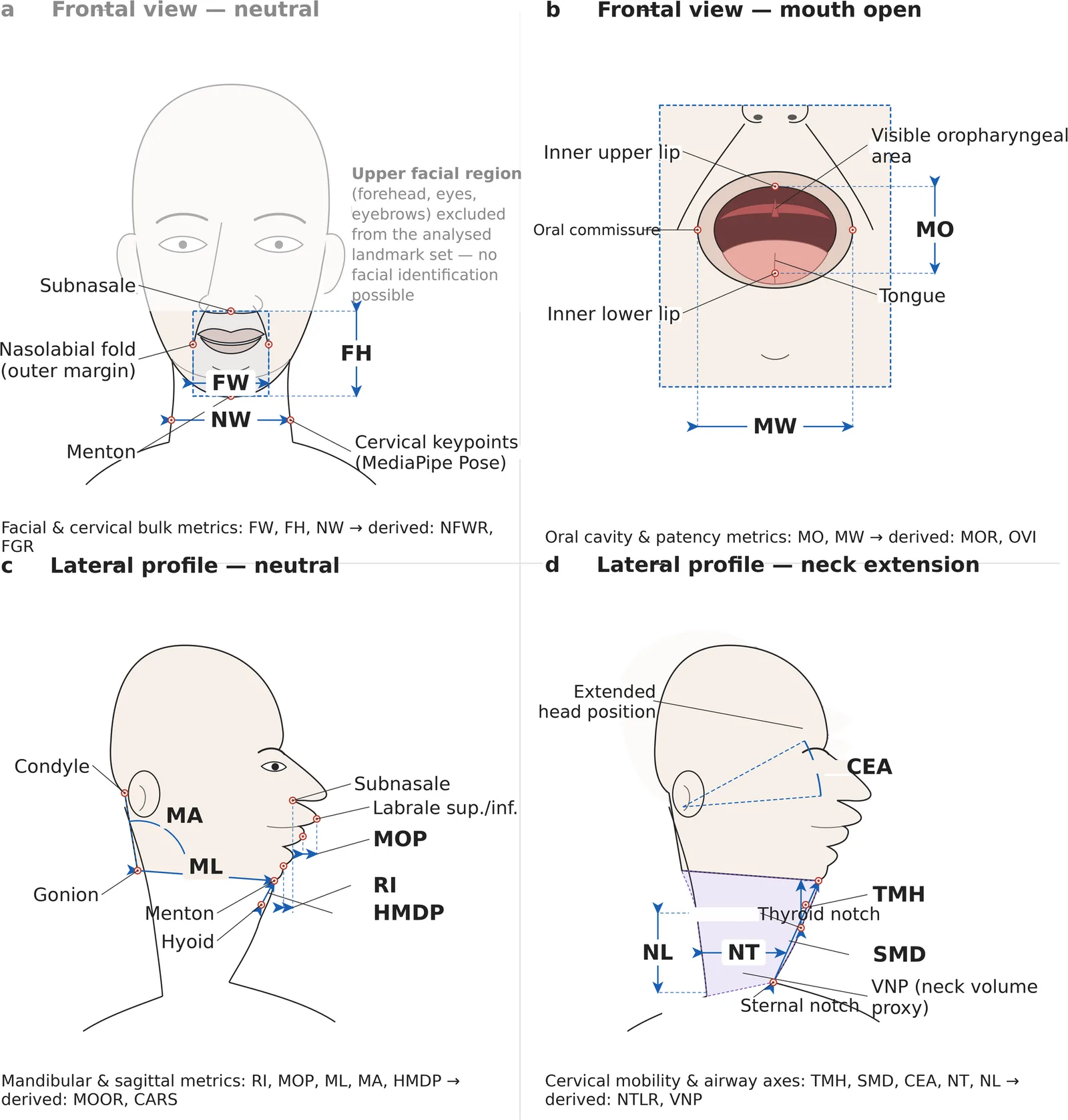
Standardized lower facial region and anatomical landmarks used for landmark-based feature extraction. Definitions, anatomical landmarks, mathematical formulations, and scale-handling strategies for all photograph-derived features are provided in Supplementary Table S1

The facial images used in this study were standardized to include only the lower facial region. The imaging field extended from the subnasal region inferiorly to the menton and was laterally bounded by the outer margins of the nasolabial folds. The forehead, eyes, eyebrows, and upper facial region were deliberately excluded from the imaging area (Fig. 2). This cropping strategy reduced the amount of directly identifying upper-facial information while preserving the lower-facial and cervical regions required for landmark extraction.

### Definition of landmark-derived anatomical features

To improve methodological transparency, Supplementary Table S1 summarizes all photograph-derived features used within the feature-extraction framework. For each feature included in the supplementary table, the corresponding photographic view, anatomical landmarks, mathematical definition, and scale-handling strategy are provided. Briefly, the extracted variables comprised direct linear measurements (e.g., facial width, neck width, mandibular length, hyomental distance proxy, thyromental height, and sternomental distance), angular measurements (e.g., mandibular angle and cervical extension angle), and normalized anatomical ratios (e.g., Mouth Opening Ratio, Neck-to-Face Width Ratio, Facial Golden Ratio, and Mandibulo-Oral Ratio). The Mouth Opening Ratio (MOR) was designed as a normalized geometric descriptor of relative mouth opening rather than an image-based estimate of the clinically measured interincisor distance and should not be interpreted as a direct surrogate for that clinical measurement. Within-image normalized ratios were designed to reduce the influence of image scale, whereas direct pixel-space measurements and scale-dependent cross-view composites were standardized during preprocessing. All measurements nevertheless depended on adherence to the standardized image-acquisition protocol.

To address the limitations of conventional pixel-based image analysis, we developed a hybrid artificial intelligence framework based on three-dimensional anatomical graph modeling and a Tabular Transformer architecture. Unlike conventional convolutional neural network (CNN)-based approaches that analyze raw pixel information, the proposed framework transforms facial and cervical images into mathematical graph representations using anatomical landmarks. This enables the extraction of clinically relevant geometric features while reducing sensitivity to image acquisition conditions such as lighting, camera distance, and background variability. For image processing and anatomical feature extraction, the open-source MediaPipe library was utilized. Traditional Convolutional Neural Networks (CNNs) process images as holistic pixel matrices, rendering them highly sensitive to background noise and illumination variability. In contrast, the MediaPipe Face Mesh architecture represents facial surface geometry using landmark-based representations, while the Pose Estimation model captures the human skeletal structure via 33 primary keypoints, together forming a high-resolution anatomical graph (3D mesh). This representation enables the extraction of Euclidean distances and geometric angles, allowing the model to focus on structural anatomy rather than superficial pixel-level information. To further enhance landmark detection robustness under variable lighting conditions, Contrast Limited Adaptive Histogram Equalization (CLAHE) was applied to normalize local illumination discrepancies prior to landmark extraction. From the extracted landmarks, clinically relevant geometric proxies—such as thyromental height, sternomental distance, and mandibular angle—were automatically derived. These photograph-derived variables were combined with the following 15 clinical and anthropometric predictors: age, sex, height, weight, body mass index (BMI), American Society of Anesthesiologists (ASA) physical status, Mallampati class, restricted interincisor opening (INCIS_TEETH), restricted neck movement (NECK_MOVE), clinically measured thyromental distance (TMD), clinically measured sternomental distance (SMD), neck circumference, clinically measured mandibular length, prominent upper-incisor appearance (RABBIT), and upper lip bite test class. The binary outcome label (TARGET), derived from the Cormack–Lehane grade, was used only as the reference outcome; neither the Cormack–Lehane grade nor TARGET was included in the predictor matrix, thereby preventing target leakage. Patient identifiers (NO and PatientID) and surgical procedure type (SURGERY) were also excluded from the predictor matrix. In addition, advanced anatomical indices, including the Retrognathia Index, Overjet Proxy, Neck Bulk Index, and Airway Visibility Index, were computed to capture subtle craniofacial and cervical characteristics associated with difficult airway anatomy. Within-image ratios derived from measurements obtained in the same standardized photograph were treated as dimensionless, whereas direct pixel-space measurements and scale-dependent or cross-view composite features were standardized during preprocessing before model development. In practical clinical imaging scenarios, landmark detection may occasionally fail due to occlusion, motion artifacts, or suboptimal image quality. To reflect this real-world limitation and avoid artificial data curation, samples with missing anatomical measurements were not excluded from analysis. Instead, missing values were handled using median imputation during preprocessing, allowing observations with partially missing landmark-derived features to be retained in the analysis. XGBoost, a gradient boosting framework known for its high performance on structured tabular data, was selected as one of the core classifiers in the ensemble [15]. Random Forest, XGBoost, stacking, and the Tabular Transformer were evaluated as candidate or comparator architectures. The final proposed hybrid model was defined as the weighted probabilistic fusion of the optimized XGBoost (40%) and Tabular Transformer (60%) outputs; Random Forest and stacking were not components of the final hybrid model. Median imputation was applied only to individual missing landmark-derived variables when the remaining image quality permitted reliable feature extraction. In contrast, patients with blurred images or images that precluded reliable landmark localization across the acquisition pipeline were excluded before analysis because meaningful feature extraction was not possible.

The Tabular Transformer architecture, originally proposed by Gorishniy et al. as a transformer-based approach specifically optimized for tabular data, consisted of a linear embedding layer projecting input features into a 32-dimensional latent space, followed by a four-head self-attention module [16].

**Fig. 3:**
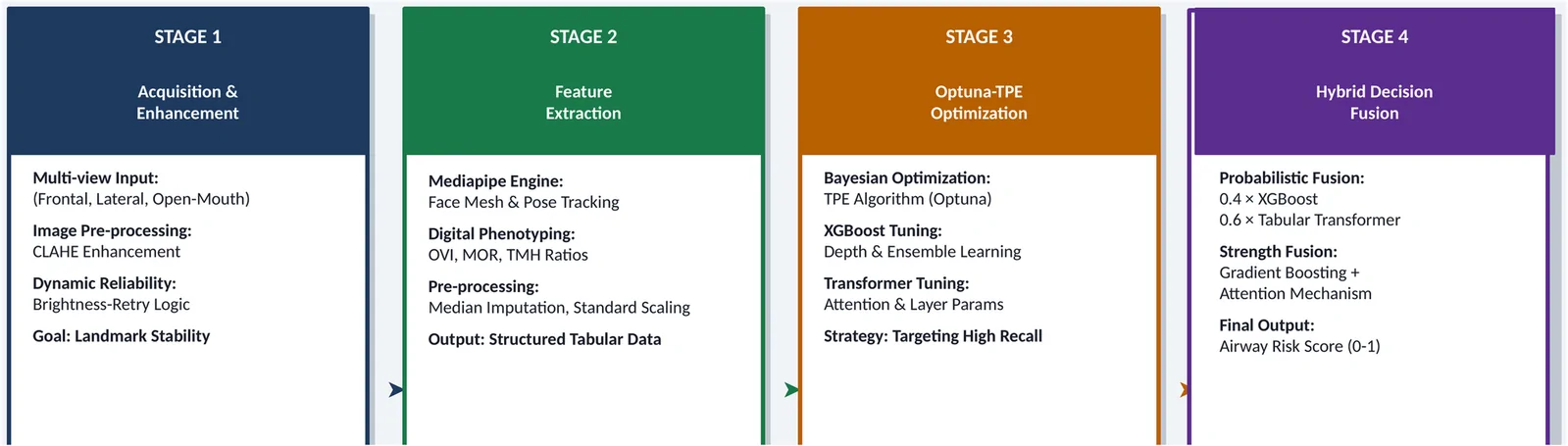
Comprehensive Workflow of the Hybrid AI System for Difficult Laryngoscopy Prediction. CLAHE, Contrast Limited Adaptive Histogram Equalization; TPE, Tree-structured Parzen Estimator; MOR, Mouth Opening Ratio; OVI, Oropharyngeal Visibility Index; SHAP, SHapley Additive exPlanations

All models evaluated in this study, including the tree-based classifiers and the Tabular Transformer, were optimized within a unified experimental framework to ensure fair and unbiased comparison. Hyperparameter optimization was conducted using the Optuna framework with a Bayesian Tree-structured Parzen Estimator (TPE) sampler, performing 30 independent optimization trials (n_trials = 30) per model [17]. The Tabular Transformer was trained using the Adam optimizer and binary cross-entropy loss, whereas the tree-based models were fitted using their respective algorithm-specific optimization procedures. To ensure strict methodological integrity and prevent data leakage, the dataset was first partitioned using a stratified 75/25 train–test split (training set: *n* = 148 [20 difficult-laryngoscopy and 128 non-difficult-laryngoscopy cases]; test set: *n* = 50 [7 difficult-laryngoscopy and 43 non-difficult-laryngoscopy cases]), preserving the distribution of difficult laryngoscopy cases (13.5% vs. 14.0%, respectively) across both subsets. All preprocessing operations, including median imputation and standard scaling, were fitted exclusively on the training data and subsequently applied to the test set using the learned parameters. Class imbalance was addressed by applying the Synthetic Minority Over-sampling Technique (SMOTE) only to the training set, ensuring that no synthetic samples influenced model evaluation [18]. Preoperative airway risk can be assessed through multiple modalities, including facial anthropometry, direct laryngoscopy, and endoscopic bronchoscopy imaging; across these modalities, structured data preprocessing and annotation quality-control steps have been shown to critically enhance the reliability of AI-based airway assessment pipelines [19]. Consistent with this principle, ratio-based features were used where anatomically appropriate, direct pixel-distance variables were standardized, and missing landmark-derived values were handled through median imputation. Model performance was assessed using a 10-fold repeated stratified cross-validation scheme with three repeats, ensuring consistent representation of the minority class across folds. The reported performance metrics were derived from the held-out test set following stratified partitioning; cross-validation was employed solely for hyperparameter optimization. To prioritize clinical safety and minimize false-negative predictions, the classification threshold was deliberately optimized at 0.40 to maximize recall (sensitivity) for difficult laryngoscopy detection. The final proposed hybrid model combined probabilistic outputs from the optimized XGBoost classifier and the Tabular Transformer using a weighted averaging strategy defined as;

$$\begin{aligned}P\left(Hybrid\right)&=0.4\:x\:p\left(XGBoost\right)\\&+0.6\:x\:p\:\left(Transformer\right)\end{aligned}$$

with greater emphasis placed on the Transformer component to enhance modeling of nonlinear feature interactions. To ensure full reproducibility, all stochastic processes were controlled using a fixed random seed across Python, NumPy, PyTorch, and system-level hash functions. Model interpretability was evaluated using SHapley Additive exPlanations (SHAP), a game-theoretic framework that quantifies each feature’s contribution to the model output [20]. A model-agnostic SHAP explainer was applied directly to the final weighted hybrid prediction function, defined as 0.40 × XGBoost probability + 0.60 × Tabular Transformer probability. A background sample of 100 observations was drawn from the SMOTE-resampled training data, and SHAP values were calculated for the held-out test set. The analysis included the complete structured feature space comprising both routinely collected clinical and anthropometric variables and photograph-derived anatomical features. (Fig. 3).

The pipeline consists of four interconnected phases:

- *Data Acquisition & Enhancement*: Raw multi-view photographs (frontal, lateral, and open-mouth) are processed using CLAHE and brightness-retry mechanisms to ensure landmark stability across varying clinical lighting conditions.
- *Automated Feature Extraction*: High-dimensional coordinates are extracted via MediaPipe Face Mesh and Pose estimators to derive clinically validated morphological indices (e.g., OVI, MOR, TMH), transforming visual data into a digital airway phenotype.
- *Intelligent Optimization & Training*: The framework utilizes the Optuna optimization engine to fine-tune both sub-architectures. Through the Tree-structured Parzen Estimator (TPE) Bayesian algorithm, the hyperparameter spaces for both the XGBoost and Tabular Transformer were systematically explored. This automated tuning ensured optimal convergence for the Transformer’s attention mechanisms and the XGBoost’s ensemble depth, specifically targeting a high Recall-rate for difficult laryngoscopy cases.
- *Hybrid Decision Fusion*: The final airway risk score is generated through a weighted probabilistic fusion (0.4 x XGBoost + 0.6 x Transformer). This hybrid approach leverages XGBoost’s efficiency in structured data patterns and the Transformer’s ability to capture complex non-linear inter-dependencies, providing a potential framework for preoperative difficult-laryngoscopy decision support.

## Results

### Demographic and clinical characteristics

**Fig. 4:**
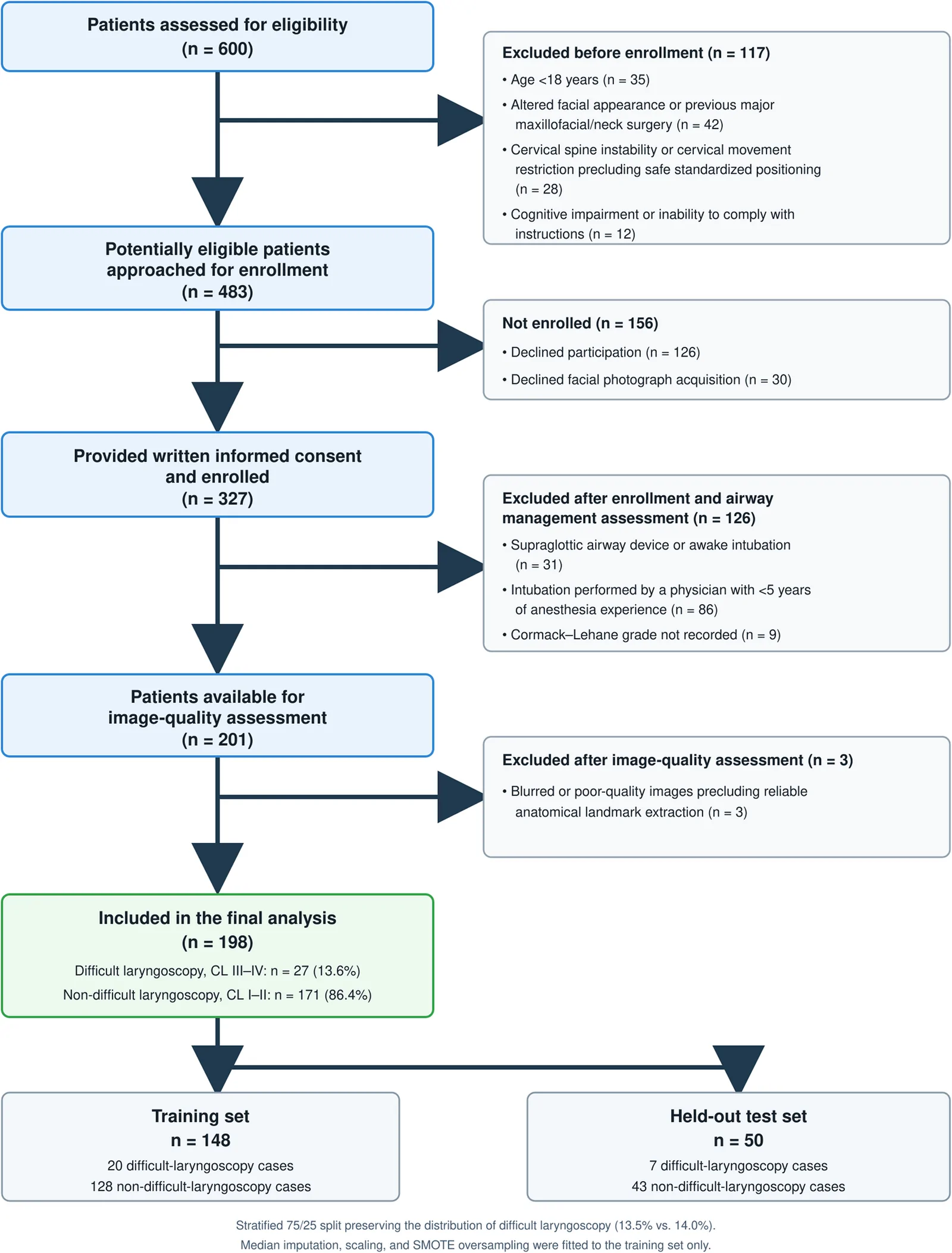
Patient Enrollment and Study Flowchart for Preoperative Airway Assessment AI Model. n, number of patients; CL, Cormack–Lehane classification; AI, artificial intelligence

A total of 600 patients scheduled for elective surgery at Gaziantep City Hospital were assessed for eligibility. Of these, 117 were excluded before enrollment because of age under 18 years (*n* = 35), altered facial appearance or previous major maxillofacial/neck surgery (*n* = 42), cervical spine instability or clinically significant restriction of cervical movement that precluded safe completion of standardized positioning maneuvers (*n* = 28), or cognitive impairment or inability to comply with the required instructions (*n* = 12). The remaining 483 potentially eligible patients were approached for enrollment. Of these, 156 were not enrolled because they declined participation (*n* = 126) or facial photograph acquisition (*n* = 30). Consequently, 327 patients provided written informed consent and were enrolled. After enrollment, 126 patients were excluded because airway management involved a supraglottic airway device or awake intubation (*n* = 31), intubation was performed by a physician with less than 5 years of anesthesia experience (*n* = 86), or the Cormack–Lehane grade was not recorded (*n* = 9). Of the remaining 201 patients, three were excluded during image-quality assessment because blurred or poor-quality images precluded reliable anatomical landmark extraction. Consequently, 198 patients were included in the final statistical analysis (Fig. 4).

The final study population consisted of 198 patients, including 101 males (51.0%) and 97 females (49.0%). According to the Mallampati classification, 70 patients (35.4%) were classified as Mallampati class I, 97 patients (49.0%) as class II, 30 patients (15.2%) as class III, and 1 patient (0.5%) as class IV. Regarding the Cormack–Lehane classification, 131 patients (66.2%) were classified as grade I, 40 patients (20.2%) as grade II, 22 patients (11.1%) as grade III, and 5 patients (2.5%) as grade IV. Accordingly, 27 patients (13.6%) were classified as difficult laryngoscopy (CL Grade III–IV), representing the minority positive class in the binary classification task. The detailed demographic and anthropometric characteristics of the study population are presented in Table 1.

**Table 1:** Baseline demographic characteristics and conventional bedside airway assessment findings of the study population

Variable	Value
Demographic characteristics	
Age (years), mean ± SD	41.14 ± 14.77
Height (cm), mean ± SD	168.14 ± 9.18
Weight (kg), mean ± SD	76.86 ± 14.44
BMI (kg/m²), mean ± SD	27.33 ± 5.67
Conventional airway assessment	
Mallampati class I	70 (35.4%)
Mallampati class II	97 (49.0%)
Mallampati class III	30 (15.2%)
Mallampati class IV	1 (0.5%)
Thyromental distance (cm), mean ± SD	9.01 ± 1.57
Sternomental distance (cm), mean ± SD	16.33 ± 2.30
Restricted interincisor opening (< 4.0 cm)	5 (2.5%)
Restricted neck movement†	19 (9.6%)
Upper lip bite test class I	172 (86.9%)
Upper lip bite test class II	25 (12.6%)
Upper lip bite test class III	1 (0.5%)

Abbreviations: *N* number of patients, *SD* standard deviation, *BMI* body mass index, *kg* kilogram, *cm* centimeter, m^2^, square meter

^†^Restricted neck movement was defined as either inability to touch the chin to the chest or cervical extension of < 30° from the neutral position among patients who could safely complete the assessment maneuvers (NECK_MOVE)

### Acquisition success rates of derived anatomical indices

Within the developed image-processing pipeline, the successful detection and acquisition rates of the automatically calculated three-dimensional geometric ratios and clinical proxy parameters derived from MediaPipe Face Mesh and Pose Estimation landmark points were evaluated. The number and percentage of cases with complete and successfully detected landmark coordinates among the total cohort of 198 patients are presented in Table 2.

**Table 2:** Landmark detection and acquisition success rates of three-dimensional anatomical and geometric indices derived from preoperative facial photographs (*n* = 198)

Feature Category / Anatomical Marker	Successful Extractions	Acquisition Rate (%)
Oral Cavity & Patency Metrics		
Mouth Opening Ratio (MOR)	193 / 198	97.5%
Oropharyngeal Visibility Index (OVI)	193 / 198	97.5%
Facial & Cervical Bulk Metrics		
Facial Width (FW)	185 / 198	93.4%
Neck Width (NW)	160 / 198	80.8%
Neck-to-Face Width Ratio (NFWR)	160 / 198	80.8%
Facial Golden Ratio (FGR)	160 / 198	80.8%
Cervical Mobility & Airway Axes		
Thyromental Height (TMH)	47 / 198	23.7%
Sternomental Distance (SMD)	44 / 198	22.2%
Cervical Extension Angle (CEA)	44 / 198	22.2%
Neck Thickness-to-Length Ratio (NTLR)	43 / 198	21.7%
Volumetric Neck Proxy (VNP)	43 / 198	21.7%
Mandibular & Sagittal Profile Metrics		
Retrognathia Index (RI)	22 / 198	11.1%
Maxillary Overjet Proxy (MOP)	22 / 198	11.1%
Mandibular Length (ML)	22 / 198	11.1%
Mandibular Angle (MA)	22 / 198	11.1%
Hyomental Distance Proxy (HMDP)	22 / 198	11.1%
Mandibulo-Oral Ratio (MOOR)	22 / 198	11.1%
Composite Anatomical Risk Score (CARS)	22 / 198	11.1%

Landmark detection and acquisition success rates are summarized in Table 2 and visually presented in Supplementary Figure S1. The highest acquisition rates were observed for the Mouth Opening Ratio (MOR) and Oropharyngeal Visibility Index (OVI), both of which were successfully extracted in 193 of 198 patients (97.5%). Facial Width (FW) was extracted in 185 patients (93.4%), while Neck Width (NW), Neck-to-Face Width Ratio (NFWR), and Facial Golden Ratio (FGR) were each extracted in 160 patients (80.8%). In contrast, lateral/profile-derived indices had lower acquisition rates, ranging from 11.1% to 23.7%, with the Retrognathia Index (RI), Maxillary Overjet Proxy (MOP), Mandibular Length (ML), Mandibular Angle (MA), Hyomental Distance Proxy (HMDP), Mandibulo-Oral Ratio (MOOR), and Composite Anatomical Risk Score (CARS) each successfully extracted in 22 patients (11.1%).

### Comparative performance of artificial intelligence models and decision fusion

All derived ratio- and coordinate-based tabular features were integrated to train Random Forest, XGBoost, and Tabular Transformer models. Following 30 independent optimization trials using the Optuna framework with a Bayesian Tree-structured Parzen Estimator (TPE) sampler, the optimal hyperparameter combinations were identified. To prioritize clinical patient safety, the classification decision threshold was intentionally optimized to 0.40, thereby minimizing false-negative predictions and maximizing recall/sensitivity performance. The comparative performance metrics of all evaluated models on the held-out test set, including accuracy, sensitivity (recall), precision, F1-score, and AUC, are presented in Table 3.

### Findings of the ablation experiment for lateral profile features

To assess the contribution of lateral/profile-derived geometric indices, including the Retrognathia Index and Mandibular Angle, which had an acquisition rate of 11.1%, an ablation analysis was performed by excluding these features from the hybrid model. After their exclusion, the AUC decreased from 0.808 to 0.787, while sensitivity (recall) for the difficult-laryngoscopy class decreased from 85.7% to 71.4%.

### Correlation analysis of high-acquisition anatomical features

Pairwise correlation analysis was performed among photograph-derived anatomical features with acquisition success rates above 20%. As shown in Supplementary Figure S2, strong correlations were observed between Facial Width and Neck Width, Mouth Opening Ratio and Oropharyngeal Visibility Index, and Neck Thickness-to-Length Ratio and Volumetric Neck Proxy. These correlations were considered descriptive and were not interpreted as measures of model-based feature importance or causal relationships.

### SHAP (SHapley Additive exPlanations) model interpretability findings

**Fig. 5:**
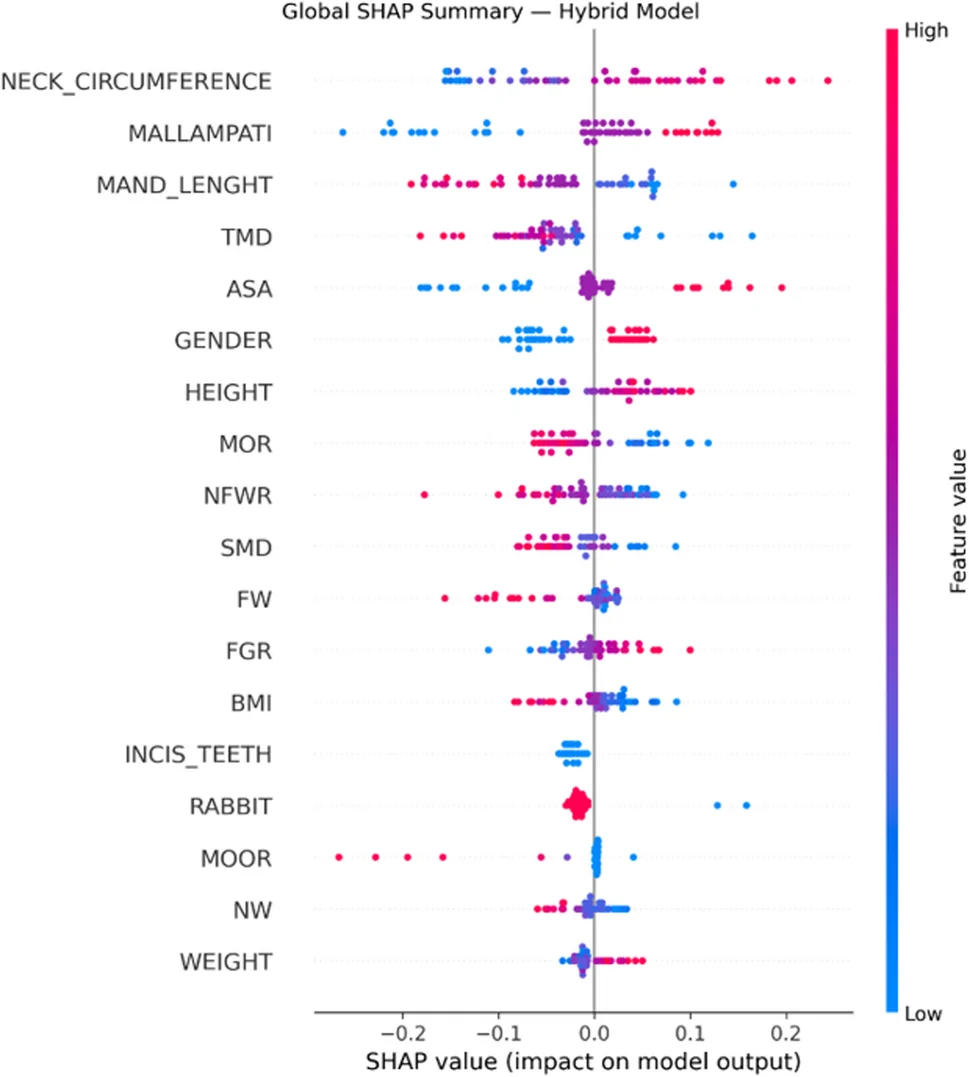
Global SHAP summary plot of the final hybrid model. Features are ranked by mean absolute SHAP value. Each point represents one held-out test-set observation; horizontal position indicates the direction and magnitude of the contribution to the predicted probability of difficult laryngoscopy, and color indicates the standardized feature value from low (blue) to high (red). SHAP, SHapley Additive exPlanations; ASA, American Society of Anesthesiologists physical status; BMI, body mass index; FGR, Facial Golden Ratio; FW, Facial Width; INCIS_TEETH, restricted interincisor opening (< 4.0 cm); MAND_LENGTH, mandibular length; MOR, Mouth Opening Ratio; MOOR, Mandibulo-Oral Ratio; NFWR, Neck-to-Face Width Ratio; NW, Neck Width; RABBIT, prominent upper-incisor appearance; SMD, Sternomental Distance; TMD, Thyromental Distance

To evaluate global model interpretability, a model-agnostic SHAP analysis was applied to the final weighted hybrid prediction function. The global SHAP beeswarm plot for the held-out test set is presented in Fig. 5, with features ranked according to their mean absolute SHAP values. Neck circumference, Mallampati classification, and mandibular length showed the greatest overall contributions to the hybrid model output, followed by thyromental distance and ASA physical status. Photograph-derived variables, including the Mouth Opening Ratio and Neck-to-Face Width Ratio, also contributed to the model predictions.

The direction and magnitude of SHAP values varied across observations, indicating nonlinear and patient-specific contributions. Higher neck circumference and higher Mallampati class generally shifted predictions toward difficult laryngoscopy, whereas greater mandibular length and thyromental distance generally shifted predictions toward non-difficult laryngoscopy. These associations describe the behavior of the fitted model and should not be interpreted as causal effects.

**Table 3:** Predictive performance of the evaluated machine learning architectures on the held-out test set (*n* = 50; 7 difficult-laryngoscopy and 43 non-difficult-laryngoscopy cases)

Model	Accuracy	Recall (Difficult)	Precision (Difficult)	F1-Score	AUC Score (95% CI)
Hybrid (Proposed Model)	80.0%	85.7%	40.0%	54.5%	0.808 (0.609–1.000)
Tabular Transformer	74.0%	71.4%	31.2%	43.5%	0.774 (0.561–0.987)
Stacking Ensemble	80.0%	28.6%	28.6%	28.6%	0.731 (0.502–0.960)
Random Forest (Optimized)	82.0%	57.1%	40.0%	47.1%	0.714 (0.487–0.941)
XGBoost (Optuna)	76.0%	71.4%	33.3%	45.5%	0.711 (0.483–0.939)

AUC 95% confidence intervals were estimated using the Hanley–McNeil method (n_positive = 7, n_negative = 43). Wilson score 95% confidence intervals for sensitivity were calculated using the Wilson score method (Hybrid AI model: 48.7–97.4%). Sensitivity and precision refer to prediction of the difficult-laryngoscopy class (Cormack–Lehane Grade III–IV). AUC, area under the receiver operating characteristic curve; CI, confidence interval; F1, harmonic mean of precision and recall

To facilitate direct comparison with routine clinical airway assessment, the diagnostic performance of the conventional bedside airway predictors routinely collected in this study and a combined clinical score derived from these variables was additionally evaluated using the identical held-out test dataset. Among the evaluated approaches, the proposed hybrid AI model demonstrated the highest overall discriminatory performance (AUC = 0.808) together with the highest sensitivity (85.7%) and negative predictive value (97.1%). Detailed comparative diagnostic performance metrics are presented in Supplementary Table S2.

**Fig. 6:**
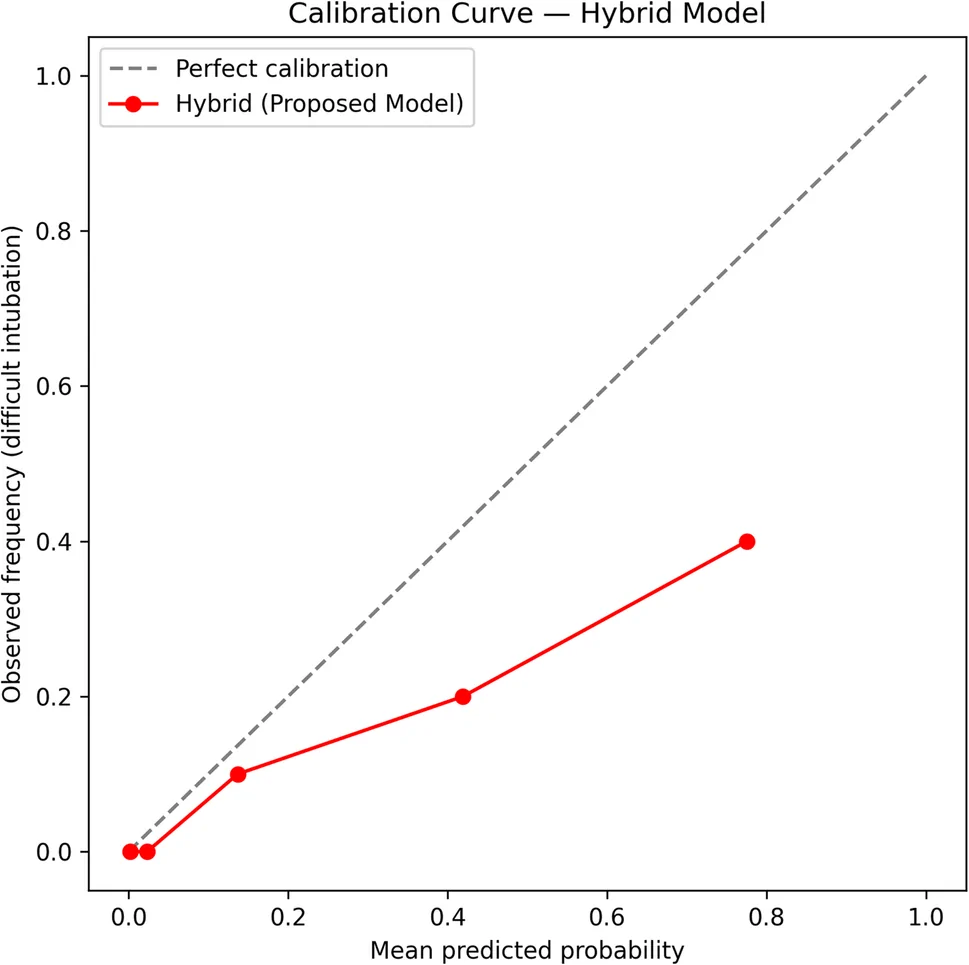
Calibration curve of the proposed hybrid AI model. The dashed diagonal line represents perfect calibration, whereas the red curve illustrates the relationship between predicted probabilities and the observed frequency of difficult laryngoscopy across probability bins

To complement discrimination-based performance measures, calibration analyses were additionally performed for all evaluated models. Calibration performance was assessed using the Brier score, calibration slope, calibration intercept, and calibration curves. For the proposed hybrid AI model, the Brier score was 0.154, the calibration slope was 0.417, and the calibration intercept was − 1.324. The corresponding calibration curve is presented in Fig. 6, whereas complete calibration statistics for all evaluated models are provided in Supplementary Table S3.

## Discussion

In this study, we developed and internally validated a multimodal hybrid AI model that integrates routinely collected clinical and anthropometric variables with landmark-derived geometric features extracted from standardized preoperative facial photographs to predict difficult laryngoscopy (Cormack–Lehane Grade III–IV) in patients undergoing elective surgery. The final hybrid architecture, based on weighted fusion of XGBoost and Tabular Transformer outputs, achieved a test-set accuracy of 80.0%, an AUC of 0.808, and a sensitivity of 85.7%. These findings support the feasibility of combining clinical information with landmark-based geometric representations for preoperative difficult-laryngoscopy risk assessment under standardized image-acquisition conditions. However, the results represent internal validation in a small, single-center cohort and require confirmation through external validation before clinical implementation.

Unexpected difficult airway management remains an important cause of severe anesthesia-related complications [21–23]. Conventional bedside airway assessment methods, including Mallampati classification, thyromental distance, and sternomental distance, have limited diagnostic performance when used individually [24–26]. Global SHAP analysis indicated that the hybrid model integrated complementary clinical, anthropometric, and photograph-derived information rather than relying on a single airway measure. In an exploratory comparison using the same held-out test set, the hybrid model showed higher discrimination than the evaluated individual bedside tests (Supplementary Table S2). However, because these bedside predictors were also included in the hybrid feature space, this comparison provides clinical context but does not establish the incremental contribution of the photograph-derived variables.

Connor and Segal demonstrated that computerized analysis of facial proportions combined with thyromental distance and Mallampati classification could identify difficult intubation with an AUROC of 0.89 and a sensitivity of 0.90 [10]. However, their method required manual placement of fiducial markers, approximately 15 min of processing per patient, and was evaluated only in Caucasian male participants. In contrast, our approach uses automated MediaPipe landmark extraction and combines standardized pixel-space measurements, normalized geometric ratios, and routinely collected clinical variables. Nevertheless, direct numerical comparison between the studies should be made cautiously because of differences in cohort composition, image acquisition, outcome definitions, and modeling strategies.

More recently, Xia et al. reported an AUC of 0.779 and a sensitivity of 0.757 using a ResNet-18 model applied to seven facial photographs from 5849 patients [27]. Other studies have also supported the feasibility of photograph-based airway assessment using interpretable deep learning and conventional machine learning methods [28–30]. Sezari et al. found that no single classical machine learning algorithm consistently outperformed the others across all performance measures, supporting further investigation of ensemble approaches [29]. Differences in populations, outcome definitions, and validation methods preclude direct comparisons across these studies. In rare-event prediction settings, overall accuracy may obscure performance in the clinically important minority class; therefore, AUC and class-specific sensitivity should be emphasized. Koca et al. reported high overall accuracy in a three-class framework, although class-specific AUC and sensitivity for the difficult-airway category were not provided [31]. Pixel-based CNN approaches, such as that used by Hayasaka et al., may also be affected by variations in illumination, background, and camera distance [3]. Su et al. recently demonstrated the feasibility of real-time facial landmark localization using an optimized YOLO11-Pose framework, although their study focused on landmark detection and Mallampati classification rather than difficult-laryngoscopy prediction [32]. These findings support the use of automated landmark extraction as a more objective alternative to subjective visual assessment while emphasizing the need for validation under heterogeneous acquisition conditions.

Three-dimensional facial scanning may provide additional anatomical depth information; however, high-fidelity implementations commonly rely on dedicated scanners and specialized acquisition and processing workflows [33, 34]. The present framework was designed to use standardized two-dimensional photographs obtained with a conventional smartphone, offering a simpler and more accessible approach. Evidence from other medical imaging applications also suggests that graph-based spatial representations may provide useful alternatives to raw pixel-based processing [35–37]. Future studies should directly compare two-dimensional landmark-based methods with three-dimensional facial imaging approaches in the same patient population.

The ablation analysis provided insight into the contribution of lateral/profile-derived features. Although frontal-view features had high acquisition success, several lateral indices were available in only a small proportion of patients because of positioning and postural limitations. Missingness was treated solely as a technical acquisition limitation and was not entered into the model as an independent predictor. Excluding the lateral/profile-derived features reduced both discrimination and sensitivity. These findings suggest that, although frontal-view features provided the principal predictive signal, lateral/profile-derived indices may offer complementary information for identifying patients at risk of difficult laryngoscopy. Future studies should determine whether systematic landmark acquisition failure itself carries additional predictive information.

SHAP values describe the behavior of the fitted model rather than causal relationships, and their interpretation is limited by the small held-out test sample. The importance of interpretable clinical features in machine learning–based airway models has also been emphasized by Kim et al. [38]. The supplementary comparison with conventional bedside predictors was exploratory, was not prespecified, and included only seven difficult-laryngoscopy cases. Moreover, established Wilson and El-Ganzouri scores could not be reconstructed because all required components were not prospectively collected. Therefore, the observed differences should not be interpreted as definitive evidence of superiority or incremental clinical value.

From a practical perspective, model inference required approximately 0.08 ms per patient; therefore, the overall workflow is expected to be determined mainly by image acquisition and landmark extraction. The use of conventional smartphone photographs may support future implementation on commonly available devices. However, prospective studies are required to determine whether image-derived features provide incremental value beyond standardized bedside assessment and established composite risk scores.

## Limitations

Despite the promising diagnostic performance of the proposed model, several limitations should be acknowledged. First, this was a single-center development and internal validation study including only elective surgical patients, which limits the generalizability of the findings. Model performance may vary across populations with different ethnic backgrounds and facial morphologies, as well as across different camera systems, image acquisition protocols, lighting conditions, and clinical environments. Consequently, external validation in larger, prospective, multicenter cohorts using heterogeneous patient populations and different imaging devices, including studies conducted in emergency and intensive care settings, is required before routine clinical implementation.

Second, the model was intentionally developed under standardized image acquisition conditions. Accordingly, patients with major craniofacial deformities, facial or neck tumors, dense facial hair, or other conditions interfering with reliable landmark detection were excluded, and the model should not yet be extrapolated to these populations.

Third, the relatively small number of difficult laryngoscopy cases in the test set resulted in wide confidence intervals and limits the precision of the reported performance estimates.

Fourth, although Cormack–Lehane grade is widely used in airway prediction research, it represents laryngoscopic view rather than the overall clinical difficulty of tracheal intubation and should therefore be regarded as a surrogate reference outcome.

Finally, the comparison with conventional bedside airway assessment methods was conducted as an exploratory supplementary analysis and was not prespecified in the original study design. In this exploratory analysis, the hybrid model demonstrated higher discrimination and sensitivity than the evaluated individual bedside predictors and the combined clinical score. Established composite risk scores, including the Wilson and El-Ganzouri scores, could not be formally reconstructed because all required components were not prospectively collected according to their original definitions. Therefore, the observed performance differences should not be interpreted as definitive evidence of superiority or incremental clinical value. Furthermore, previous difficult airway history and detailed procedural airway management variables, including videolaryngoscope use, number of intubation attempts, and airway adjunct use, were not prospectively collected in a standardized manner. Future prospective studies should perform prespecified head-to-head comparisons of the proposed model with standardized bedside airway assessment methods and formally calculated composite airway risk scores. In addition, repeated-image reproducibility and intra-subject landmark variability were not evaluated in the present study and should be investigated in future prospective validation studies.

## Conclusion

In conclusion, the proposed hybrid artificial intelligence approach integrated routinely collected clinical and anthropometric variables with MediaPipe-derived geometric features through weighted fusion of Tabular Transformer and XGBoost outputs. The model demonstrated encouraging internal discrimination and sensitivity for preoperative difficult-laryngoscopy risk assessment, while SHAP analysis provided insight into the contributions of clinical and photograph-derived features. External validation in larger and more heterogeneous populations, together with prespecified evaluation of the incremental contribution of image-derived features beyond standardized clinical assessment and established composite risk scores, is required before routine clinical implementation. At present, the model should be regarded as a complementary decision-support tool rather than a replacement for conventional preoperative airway assessment.

## Supplementary Information

Supplementary Material 1.

Supplementary Material 2.

Supplementary Material 3.

Supplementary Material 4.

Supplementary Material 5.

Supplementary Material 6.

## Data Availability

The datasets generated and analysed during the current study contain identifiable facial photographs and are not publicly available due to patient privacy regulations and ethical constraints. De-identified tabular feature data may be made available upon reasonable request to the corresponding author, subject to institutional approval.
