# Supplementary material for: Predicting difficult laryngoscopy via three-dimensional graphical modeling and hybrid artificial intelligence using standard facial photographs in elective surgery patients
Source: BMC Anesthesiol. 2026 Aug 15;26:614. doi: 10.1186/s12871-026-04160-9 (PMC13660001; doi:10.1186/s12871-026-04160-9)
Supplement: Supplementary file 1 [file 12871_2026_4160_MOESM1_ESM.docx]

**Supplementary Table S1. Code-verified definitions, landmark indices, mathematical formulations, and scale-handling strategies for the photograph-derived features used in the hybrid model.**

| **Feature (abbreviation) / code variable** | **Source photograph** | **Landmarks** | **Code-verified formula** | **Scale handling and interpretation** |
| --- | --- | --- | --- | --- |
| **Retrognathia Index (RI) / IMG_Retrognathia_Index** | head_normal_side | Face Mesh L₁₅₂ and L₀; horizontal coordinates only | RI = \|x₁₅₂ − x₀\|W | Pixels; scale-dependent; standardized before fitting; horizontal-offset proxy |
| **Maxillary Overjet Proxy (MOP) / IMG_Overjet_Proxy** | head_normal_side | Face Mesh L₀ and L₁₇; horizontal coordinates only | MOP = \|x₀ − x₁₇\|W | Pixels; scale-dependent; standardized before fitting; horizontal-offset proxy |
| **Mandibular Length (ML) / IMG_Mandibula** | head_normal_side | Face Mesh L₁₅₂ and L₂₃₄ | ML = d(L₁₅₂,L₂₃₄) | Pixels; scale-dependent; standardized before fitting |
| **Mandibular Angle (MA) / IMG_Mandibular_Angle** | head_normal_side | Vertex L₁₅₂; synthetic horizontal point (1,y₁₅₂); L₂₃₄ | MA = θ[(1,y₁₅₂),L₁₅₂,L₂₃₄] | Degrees in normalized coordinate space; computational orientation index, not a calibrated anatomical angle |
| **Hyomental Distance Proxy (HMDP) / IMG_Hyomental_Proxy** | head_normal_side | Face Mesh L₁₅₂ and L₁₇₂ | HMDP = d(L₁₅₂,L₁₇₂) | Pixels; scale-dependent; standardized before fitting; landmark proxy |
| **Thyromental Height Proxy (TMH) / IMG_Thyromental_Height_Proxy** | head_normal_side (Pose) | Pose P₀ and shoulder midpoint S=(P₁₁+P₁₂)/2 | TMH = \|y₀ − yS\|H | Pixels; vertical pose-derived proxy; not direct clinical thyromental height; standardized before fitting |
| **Facial Width (FW) / IMG_FaceWidth** | head_normal_front | Face Mesh L₂₃₄ and L₄₅₄ | FW = d(L₂₃₄,L₄₅₄) | Pixels; scale-dependent; standardized before fitting |
| **Neck Width Proxy (NW) / IMG_NeckWidthProxy** | head_normal_front (Pose) | Pose P₁₁ and P₁₂ | NW = d(P₁₁,P₁₂) | Pixels; shoulder-keypoint-derived width proxy; standardized before fitting |
| **Neck-to-Face Width Ratio (NFWR) / RATIO_Neck_Bulk_Index** | head_normal_front | Derived from NW and FW | NFWR = NW/FW | Dimensionless ratio; scale-invariant when both components are obtained from the same standardized view |
| **Sternomental Distance Proxy (SMD) / IMG_Sternomental** | neck_extended_side (Pose) | Pose P₀ and shoulder midpoint S=(P₁₁+P₁₂)/2 | SMD = d(P₀,S) | Pixels; pose-derived cranio-shoulder distance proxy; not direct clinical sternomental distance; standardized before fitting |
| **Cervical Extension Angle (CEA) / IMG_Neck_Extension_Angle** | neck_extended_side (Pose) | P₀, shoulder midpoint S, and synthetic vertical point V=(xS,1) | CEA = θ(P₀,S,V) | Degrees in normalized coordinate space; computational orientation index, not a calibrated physical angle |
| **Mouth Opening Ratio (MOR) / IMG_Mouth_Open_Ratio** | mouth_open_front | L₁₃, L₁₄, L₆₁, and L₂₉₁ | MOR = d(L₁₃,L₁₄)/d(L₆₁,L₂₉₁) | Dimensionless ratio; relative inter-lip opening, not clinical interincisor distance |
| **Oropharyngeal Visibility Index (OVI) / IMG_Airway_Visibility_Index** | mouth_open_front | L₁₃, L₁₄, L₁₀, and L₁₅₂ | OVI = d(L₁₃,L₁₄)/d(L₁₀,L₁₅₂) | Dimensionless ratio; scale-invariant within the same image |
| **Facial Golden Ratio (FGR) / RATIO_Facial_Golden** | head_normal_front | Derived from FW and NW | FGR = FW/NW | Dimensionless ratio; reciprocal of NFWR when both values are available before imputation |
| **Neck Thickness-to-Length Ratio (NTLR) / RATIO_Neck_Thick_Length** | head_normal_front + neck_extended_side | Derived from NW and SMD | NTLR = NW/SMD | Dimensionless cross-view ratio; depends on standardized acquisition across views |
| **Mandibulo-Oral Ratio (MOOR) / RATIO_Mandibulo_Oral_Opening** | head_normal_side + mouth_open_front | Derived from MOR and ML | MOOR = MOR/ML | Inverse-pixel composite; scale-dependent because ML is pixel-based; standardized before fitting |
| Composite Anatomical Risk Score (CARS) / RATIO_Anatomical_Risk_Combo | head_normal_side + mouth_open_front | Derived from RI and OVI | CARS = RI(1−OVI) | Pixel-scaled composite; scale-dependent through RI; standardized before fitting |
| Volumetric Neck Proxy (VNP) / RATIO_Neck_Volume_Proxy | head_normal_front + neck_extended_side | Derived from NFWR and SMD | VNP = NFWR/SMD | Inverse-pixel composite; scale-dependent through SMD; standardized before fitting |

All features were extracted from standardized photographs using MediaPipe Face Mesh and Pose. Let Lᵢ=(xᵢ,yᵢ) denote Face Mesh landmark i and Pᵢ=(xᵢ,yᵢ) denote Pose landmark i, with normalized coordinates in [0,1]. For an image of height H and width W, the pixel-space Euclidean distance is d(A,B)=√{[(xA−xB)W]²+[(yA−yB)H]²}. For three normalized-coordinate points A, B, and C, with B as the vertex, θ(A,B,C)=arccos{[(A−B)·(C−B)]/[‖A−B‖‖C−B‖]} in degrees. Direct pixel-based features and scale-dependent composite features were standardized (z-scored) after training-set median imputation; dimensionless ratios were retained as calculated.

The feature names TMH, SMD, HMDP, RI, and MOP denote computational landmark-derived proxies and should not be interpreted as direct equivalents of manually measured clinical airway distances. OVI is internally implemented as IMG_Airway_Visibility_Index. Angles were calculated in normalized coordinate space; MA and CEA use synthetic horizontal or vertical reference points and therefore represent computational orientation indices rather than calibrated physical angles.
