# Supplementary material for: Predicting difficult laryngoscopy via three-dimensional graphical modeling and hybrid artificial intelligence using standard facial photographs in elective surgery patients
Source: BMC Anesthesiol. 2026 Aug 15;26:614. doi: 10.1186/s12871-026-04160-9 (PMC13660001; doi:10.1186/s12871-026-04160-9)
Supplement: Supplementary file 2 [file 12871_2026_4160_MOESM2_ESM.docx]

**Supplementary Table S2**

| **Predictor** | **Accuracy** | **Sensitivity** | **Specificity** | **PPV** | **NPV** | **AUC** |
| --- | --- | --- | --- | --- | --- | --- |
| Mallampati classification | 0.780 (0.660–0.880) | 0.429 (0.000–0.833) | 0.837 (0.721–0.933) | 0.300 (0.000–0.601) | 0.900 (0.800–0.976) | 0.725 (0.565–0.881) |
| Upper Lip Bite Test | 0.860 (0.760–0.940) | 0.000 (0.000–0.000) | 1.000 (1.000–1.000) | — | 0.860 (0.760–0.940) | 0.534 (0.433–0.696) |
| Thyromental distance | 0.780 (0.640–0.881) | 0.286 (0.000–0.668) | 0.860 (0.744–0.954) | 0.250 (0.000–0.625) | 0.881 (0.773–0.975) | 0.600 (0.366–0.833) |
| Sternomental distance | 0.781 (0.660–0.900) | 0.286 (0.000–0.668) | 0.861 (0.750–0.953) | 0.259 (0.000–0.600) | 0.881 (0.775–0.976) | 0.699 (0.486–0.886) |
| Combined clinical score | 0.620 (0.480–0.760) | 0.714 (0.333–1.000) | 0.605 (0.462–0.750) | 0.227 (0.056–0.412) | 0.929 (0.818–1.000) | 0.769 (0.591–0.916) |
| Hybrid AI model | 0.800 (0.680–0.900) | 0.857 (0.500–1.000) | 0.791 (0.666–0.905) | 0.400 (0.154–0.667) | 0.971 (0.906–1.000) | 0.808 (0.644–0.932) |

**Supplementary Table S2. Comparative diagnostic performance of conventional bedside airway assessment methods, the combined clinical score, and the proposed hybrid AI model using the identical held-out test dataset.**

**Abbreviations:** PPV, positive predictive value; NPV, negative predictive value; AUC, area under the receiver operating characteristic curve. All metrics were calculated using the identical held-out test dataset.
