# Supplementary material for: Predicting difficult laryngoscopy via three-dimensional graphical modeling and hybrid artificial intelligence using standard facial photographs in elective surgery patients
Source: BMC Anesthesiol. 2026 Aug 15;26:614. doi: 10.1186/s12871-026-04160-9 (PMC13660001; doi:10.1186/s12871-026-04160-9)
Supplement: Supplementary file 3 [file 12871_2026_4160_MOESM3_ESM.docx]

**Supplementary Table S3. Calibration performance of all evaluated machine-learning models on the held-out test dataset.**

| **Model** | **Brier score** | **Calibration slope** | **Calibration intercept** |
| --- | --- | --- | --- |
| **Random Forest** | 0.138 | 0.635 | -1.170 |
| **XGBoost** | 0.171 | 0.185 | -1.475 |
| **Stacking** | 0.141 | 0.443 | -1.050 |
| **Tabular Transformer** | 0.176 | 0.333 | -1.373 |
| **Hybrid (Proposed)** | 0.154 | 0.417 | -1.324 |

Calibration performance of the evaluated machine-learning models on the held-out test dataset (n = 50). Calibration was assessed using the Brier score, calibration slope, and calibration intercept. Lower Brier scores indicate better overall probabilistic accuracy, whereas calibration parameters closer to their ideal values (slope = 1 and intercept = 0) indicate better agreement between predicted probabilities and observed outcomes.
