# Supplementary material for: Predicting difficult laryngoscopy via three-dimensional graphical modeling and hybrid artificial intelligence using standard facial photographs in elective surgery patients
Source: BMC Anesthesiol. 2026 Aug 15;26:614. doi: 10.1186/s12871-026-04160-9 (PMC13660001; doi:10.1186/s12871-026-04160-9)
Supplement: Supplementary file 4 [file 12871_2026_4160_MOESM4_ESM.docx]

**Supplementary Table S4**

**Supplementary Table S4. Diagnostic performance of all evaluated machine-learning models on the held-out test dataset with 95% confidence intervals.**

| **Model** | **Accuracy (95% CI)** | **Sensitivity (95% CI)** | **Specificity (95% CI)** | **PPV (95% CI)** | **NPV (95% CI)** | **AUC (95% CI)** |
| --- | --- | --- | --- | --- | --- | --- |
| Random Forest | 0.820 (0.719–0.920) | 0.571 (0.167–1.000) | 0.860 (0.750–0.955) | 0.400 (0.100–0.727) | 0.925 (0.838–1.000) | 0.714 (0.487–0.941) |
| XGBoost (Optuna) | 0.760 (0.640–0.880) | 0.714 (0.333–1.000) | 0.767 (0.638–0.889) | 0.333 (0.100–0.583) | 0.943 (0.857–1.000) | 0.711 (0.483–0.939) |
| Stacking Ensemble | 0.800 (0.680–0.900) | 0.286 (0.000–0.668) | 0.884 (0.778–0.975) | 0.286 (0.000–0.667) | 0.884 (0.780–0.976) | 0.731 (0.502–0.960) |
| Tabular Transformer | 0.740 (0.620–0.860) | 0.714 (0.286–1.000) | 0.744 (0.609–0.872) | 0.313 (0.083–0.556) | 0.941 (0.848–1.000) | 0.774 (0.561–0.987) |
| Hybrid (Proposed Model) | 0.800 (0.680–0.900) | 0.857 (0.500–1.000) | 0.791 (0.666–0.905) | 0.400 (0.154–0.667) | 0.971 (0.906–1.000) | 0.808 (0.609–1.000) |

Supplementary Table S4. Diagnostic performance of all evaluated machine-learning models on the held-out test dataset with 95% confidence intervals. Confidence intervals for accuracy, sensitivity, specificity, positive predictive value (PPV), and negative predictive value (NPV) were estimated using bootstrap resampling. The 95% confidence intervals for AUC were calculated using the Hanley–McNeil method.
