# Supplementary material for: Predicting difficult laryngoscopy via three-dimensional graphical modeling and hybrid artificial intelligence using standard facial photographs in elective surgery patients
Source: BMC Anesthesiol. 2026 Aug 15;26:614. doi: 10.1186/s12871-026-04160-9 (PMC13660001; doi:10.1186/s12871-026-04160-9)
Supplement: Supplementary file 5 [file 12871_2026_4160_MOESM5_ESM.docx]

**Supplementary Figure S1.** Landmark detection and acquisition success rates of three-dimensional anatomical and geometric indices derived from preoperative facial photographs (n=198). High acquisition rates were observed for frontal-view parameters, whereas lateral/profile-derived indices had lower acquisition rates, ranging from 11.1% to 23.7%.
