# Supplementary material for: Predicting difficult laryngoscopy via three-dimensional graphical modeling and hybrid artificial intelligence using standard facial photographs in elective surgery patients
Source: BMC Anesthesiol. 2026 Aug 15;26:614. doi: 10.1186/s12871-026-04160-9 (PMC13660001; doi:10.1186/s12871-026-04160-9)
Supplement: Supplementary file 6 [file 12871_2026_4160_MOESM6_ESM.docx]

**Supplementary Figure S2.** Correlation heatmap of the photograph-derived anatomical features with acquisition success rates above 20%. Color intensity represents the direction and magnitude of pairwise correlation coefficients. The final row and column display unadjusted correlations with difficult laryngoscopy and should not be interpreted as measures of model-based feature importance or causal association.
